# Obituary

**Published:** 1909-10-15

**Authors:** 


### OBITUARY.

### DOCTOR FREDERICK SHIVELY WHITSLAR.

Doctor Frederick Shively Whitslar of Youngstown, Ohio, died from a stroke of paralysis resulting from emphysema of the lungs with heart complications on August 7th, 1909.

Dr. Whitslar was born in Austintown township, then Trumbull Co., O., on September 7th, 1824. In March 1849 he was married to Miss Matilda Fox, who died October 28, 1898. Three children, Dr. W. H. Whitslar of Cleveland, Grant S. Whitslar, of Youngstown, and Mrs. Allie Carr, who made her home with her father, survive him. A sister, Mrs. Darrow, of Youngstown, is also living.

The deceased was a self-made man in every respect. Born in pioneer days of Ohio he was bound out as a youth to work on a farm. While a young man he taught school in the old log school houses and later took up the practice of dentistry. He commenced his profession by studying Harris's Principles and Practice, received some instruction from Dr. Corydon Palmer, and learned the rest in practice and dental societies. He engaged in the practice of dentistry over fifty years, writing many articles for dental magazines which gave him a national reputation. He was well known professionally and highly esteemed.

From Ambler's "Tin Foil," page 99, we find that Dr. Whitslar was the first to use tin as a filling material in 1845.

He was active in politics, especially just before the civil war, when he was an anti-slavery advocate and assisted many slaves to escape by the underground railway. He was the first president of the Youngstown city council.

When the strife between the north and south arose he enlisted to serve his country.

He was a captain in the civil war and performed gallant and meritorious service for which he was commended by his superior officers for his bravery and conduct. He received an honorable discharge at Camp Dennison on the 27th day of August 1864.

He was elder of the Central Christian church of Youngstown and often preached for ministers of other denominations. He was an honored man and highly esteemed by all who knew him.

Dr. Whitslar was a member of Tod Post No. 29, Department of Ohio, Grand Army of Republic. Also a member of the Delta Sigma Delta fraternity; The American Dental Association, the Northern Ohio Dental Association, of which he was once president; Mahoning Co. Dental Associa tion; Odontological Society of W. Pa.; Twenty-second District Missionary Society, of which he was president, and charter member, and organizer of the City Library Association.

